# Pharmacokinetic differences in nicotine and nicotine salts mediate reinforcement-related behavior: an animal model study

**DOI:** 10.3389/fnins.2023.1288102

**Published:** 2023-11-16

**Authors:** Pengfei Han, Xiaoyuan Jing, Shulei Han, Xinsheng Wang, Qiannan Li, Yuan Zhang, Pengpeng Yu, Xin-an Liu, Ping Wu, Huan Chen, Hongwei Hou, Qingyuan Hu

**Affiliations:** ^1^Institute of Biomedical Engineering, College of Life Sciences, Qingdao University, Qingdao, China; ^2^China National Tobacco Quality Supervision and Test Center, Zhengzhou, China; ^3^Key Laboratory of Tobacco Biological Effects, Zhengzhou, China; ^4^Beijing Life Science Academy, Beijing, China; ^5^Key Laboratory of Tobacco Biological Effects and Biosynthesis, Beijing, China; ^6^Shenzhen Institute of Advanced Technology, Chinese Academy of Sciences, Shenzhen, China; ^7^National Institute on Drug Dependence and Beijing Key Laboratory of Drug Dependence, Beijing, China

**Keywords:** nicotine salts, self-administration, pharmacokinetics, dopamine, anxiety

## Abstract

Since their introduction in the United States and Europe in 2007, electronic cigarettes (E-Cigs) have become increasingly popular among smokers. Nicotine, a key component in both tobacco and e-cigarettes, can exist in two forms: nicotine-freebase (FBN) and nicotine salts (NS). While nicotine salt is becoming more popular in e-cigarettes, the effect of nicotine salts on reinforcement-related behaviors remains poorly understood. This study aimed to compare the reinforcing effects of nicotine and nicotine salts in animal models of drug self-administration and explore potential mechanisms that may contribute to these differences. The results demonstrated that three nicotine salts (nicotine benzoate, nicotine lactate, and nicotine tartrate) resulted in greater reinforcement-related behaviors in rats compared to nicotine-freebase. Moreover, withdrawal-induced anxiety symptoms were lower in the three nicotine salt groups than in the nicotine-freebase group. The study suggested that differences in the pharmacokinetics of nicotine-freebase and nicotine salts *in vivo* may explain the observed behavioral differences. Overall, this study provides valuable insights into the reinforcing effects of nicotine as well as potential differences between nicotine-freebase and nicotine salts.

## 1. Introduction

Electronic cigarettes (E-Cigs) have increased in popularity among smokers since their inception in the United States and Europe in 2007 (Mirbolouk et al., 2018). Nicotine is the most pharmacologically active component in tobacco products, and it is an important component of electronic cigarettes (Benowitz, 2010; Gholap et al., 2020). Nicotine is an alkaloid which can be separated as a freebase, but when mixed with an organic acid, it is protonated and produces a salt. The nicotine-freebase (FBN) was commonly employed in early e-Cig liquids. However, in recent years, more e-cigarettes, such as JUUL, have used nicotine-salts (NS) in their e-Cig liquids (Harvanko et al., 2020).

Currently, nicotine-salt research is focused on pharmacokinetics (Benowitz, 2009; O’Connell et al., 2019) and sensory evaluation (Leventhal et al., 2021; Han et al., 2023). Experiments on reinforcement-related behavior can enable animals to associate specific operant behaviors with rewards throughout the process of drug research, such as self-administration experiments, which can simulate human drug abuse behavior and directly examining the reinforcement effect of drugs (Funada, 2010).

Nonetheless, there have been few reports on the effect of nicotine salts on reinforcement-related behavior. In vapor self-administration, nicotine-salt produces greater reinforcement-related behaviors in male and female mice when compared to nicotine-freebase (Henderson and Cooper, 2021). However, this report only included one nicotine-salt (nicotine tartrate; NT, the most commonly use nicotine salt in prior studies), while the two most prevalent nicotine salts (NB; nicotine benzoate, NL; nicotine lactate) in e-liquids (Harvanko et al., 2020) were omitted. It is difficult to precisely control the intake of animals in vapor self-administration experiments.

Currently, preclinical experiments of intravenous self-administration in rodents have provide the majority of neuronal understanding of nicotine (Hawk Jr. et al., 2015; McKee et al., 2016; Chukwueke et al., 2020). The intravenous self-administration model directly explains the relationship between subjective effects induced by drugs and drug-seeking behaviors, and the drug dose obtained each time is more precise (Donny et al., 1998).

According to prior studies, nicotine metabolism is closely related to reinforcement-related behavior. The number of nicotine self-administrations in mice decreased significantly after addition of the CYP2A5 inhibitor DLCI-1 (Chen et al., 2020), another study demonstrated a lack of nicotine enhancing behavior in mice conditioned place preference (CPP) tests after adding CYP2A5 inhibitors (Bagdas et al., 2014).

Our group has demonstrated that the peak plasma nicotine concentration of nicotine-freebase was approximately two times higher than nicotine salts, and the Tmax of nicotine salts was shorter than that of nicotine-freebase at a dose of 1 mg/kg nicotine (Han et al., 2022). Therefore, we speculate that reinforcement-related behaviors may be affected by the pharmacokinetics of nicotine-freebase and nicotine salts *in vivo*.

Nicotine stimulates increases in extracellular dopamine (DA) through activation of dopamine neurons, resulting in reward and reinforcement (Rice and Cragg, 2004; De Biasi and Dani, 2011). It is suggested that the increase in DA level in the midbrain induced by nicotine is an important indicator of reinforcement-related behavior analysis (Barrett et al., 2004; Brody et al., 2004; di Chiara et al., 2004; De Biasi and Dani, 2011).

In this study, the three most used nicotine salts (nicotine benzoate, nicotine lactate, and nicotine tartrate) were selected for intravenous self-administration experiments in rats. Next, open field test (OFT) and elevated plus-maze (EPM) experiments were used to determine behavioral differences during nicotine withdrawal. Additional pharmacokinetic experiments and desorption electrospray ionization-mass spectrometry imaging (DESI-MSI) experiments were conducted to detect the metabolism of nicotine-freebase and nicotine salts in the blood and distribution of nicotine in the brain, respectively. Microdialysis experiments were conducted to detect the dopamine release caused by nicotine-freebase and nicotine salts in the nucleus accumbent (NAc). Quantitative Real-time Polymerase Chain Reaction (RT-qPCR) experiments were conducted to detect expression of nicotine acetylcholine receptors within the ventral tegmental area (VTA) caused by administration of nicotine-freebase and nicotine salts. Ultimately, the potential mechanisms underlying observed behavioral differences were determined.

## 2. Materials and methods

### 2.1. Study design

#### 2.1.1. Self-administration and withdrawal-related behaviors

**Figure 1:**
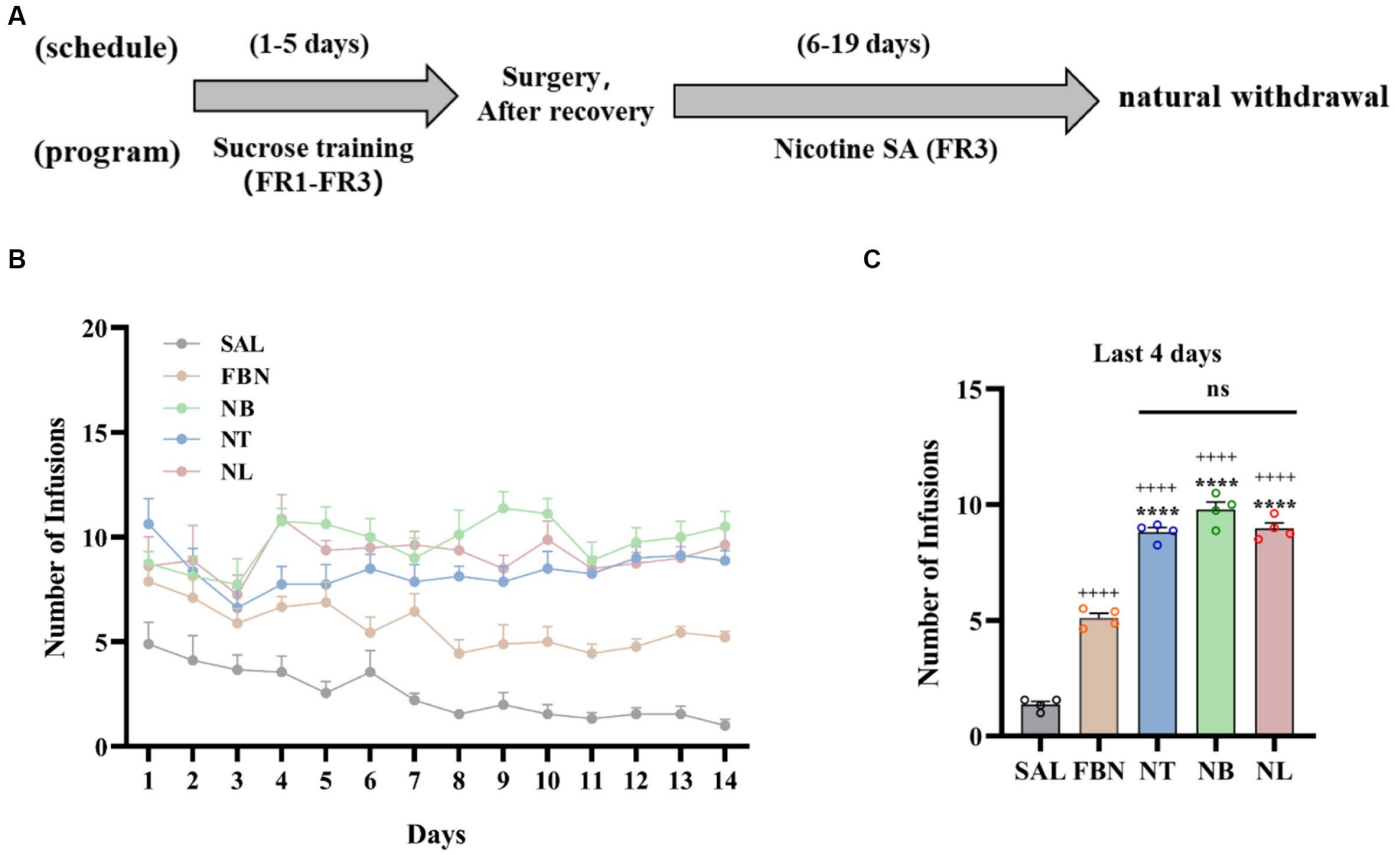
Results of self-administration experiments under a fixed-rate 3 (FR3) schedule. **(A)** Experimental timeline. **(B)** Number of infusions for each group: saline (SAL, black circle), nicotine-freebase (FBN, yellow circle), nicotine tartrate (NT, blue circle), nicotine benzoate (NB, green circle), and nicotine lactate (NL, pink circle). **(C)** Average number of infusions in the last 4 days of rats in each group. Error bars represent the mean ± SEM (*n* = 8). Bonferroni Student-*t* test after ANOVAS: *p < 0.05, **p < 0.01, ***p < 0.001, and ****p < 0.0001 versus FBN group; ^++++^*p* < 0.0001 versus SAL group.

These experiments (see details in Figure 1A) utilized 40 SD rats randomly divided into five groups (saline, nicotine-freebase, nicotine tartrate, nicotine benzoate, nicotine lactate), with 8 rats in each group. The dose administered in the self-administration experiments was 0.03 mg/kg (nicotine doses are calculated as free base). An SA experiment was used to assess reinforcement-related behaviors, open field test (OFT) was used to test locomotion in rats, and an elevated plus-maze (EPM) test was used to monitor anxiety-like behavior in rats.

#### 2.1.2. Withdrawal-related behaviors under the same dose administration

**Figure 2:**
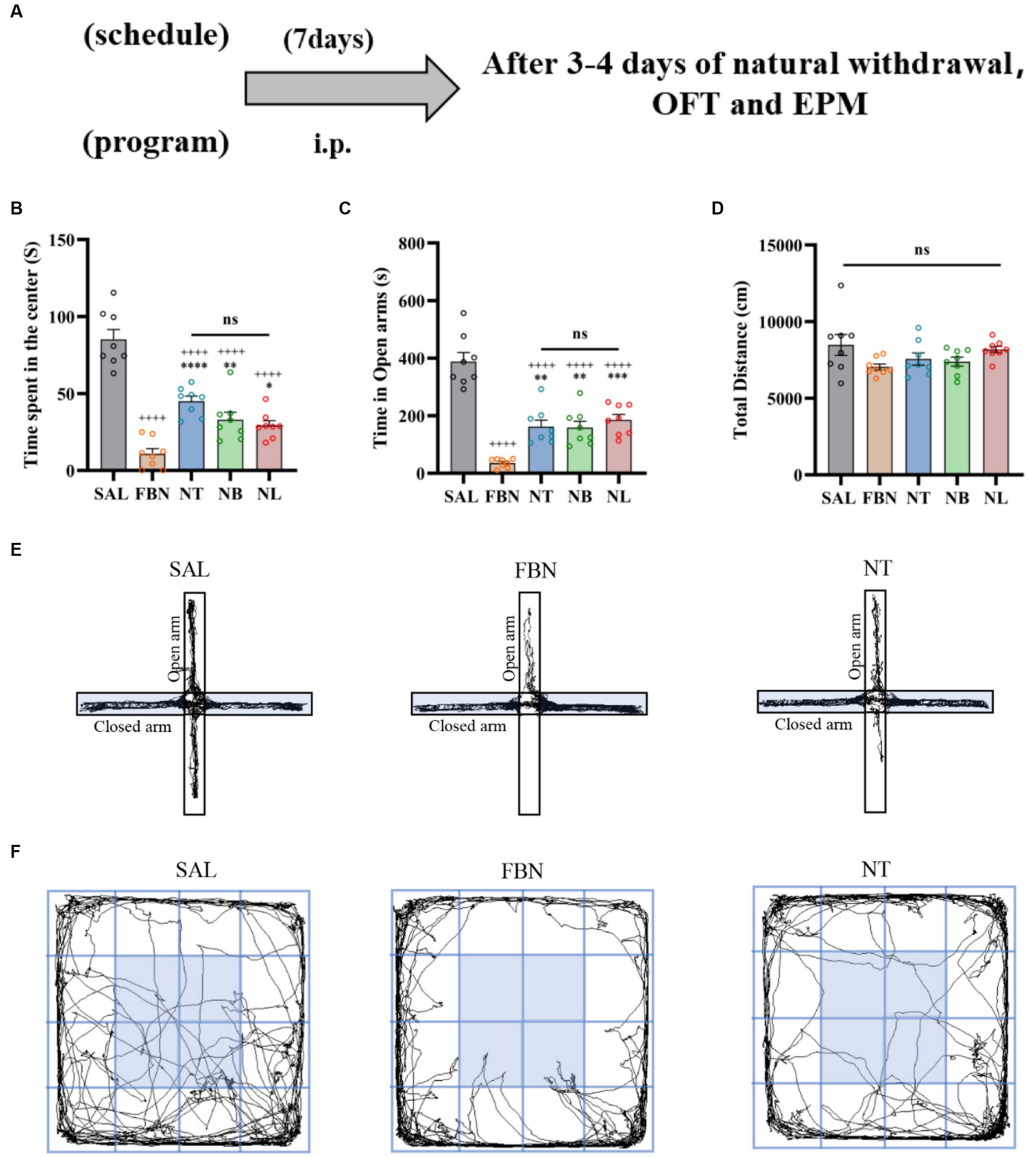
Results of the EPM and OFT tests conducted after 7 days of intraperitoneal injection and 3–4 days of natural withdrawal. **(A)** Timeline of withdrawal-related behavioral experiments. **(B)** Time spent in the center. **(C)** Open arm duration. **(D)** Total distance traveled. **(E)** Representative images of each group in the EPM. **(F)** Representative images of each group in the OFT. Only one representative image is shown for nicotine salt groups, as there were no significant differences in withdrawal-related behaviors between these groups. Bonferroni Student-*t* test after ANOVAS: *p < 0.05, **p < 0.01, and ***p < 0.001 versus FBN group; ^++++^ *p* < 0.0001 versus SAL group (*n* = 8).

To distinguish differences in withdrawal-related behaviors between the nicotine-freebase group and the nicotine salts group, OFT and EPM tests were performed on individuals receiving the same dose administration. The experiments were conducted on 40 SD rats, which were randomly allocated into five groups (saline, nicotine-freebase, nicotine tartrate, nicotine benzoate, nicotine lactate) with 8 rats in each group. Each rat received an intraperitoneal (i.p.) injection of 0.5 mg/kg nicotine-freebase or nicotine salts twice daily, this choice of method and dose has been reported in previous studies establishing rat models of nicotine addiction (Chellian et al., 2021). Three to 4 days after the natural withdrawal period, EPM and OFT tests were conducted. The procedures for the EPM and OFT experiments are shown in Figure 2A.

#### 2.1.3. Pharmacokinetics of self-administration related doses

**Figure 3:**
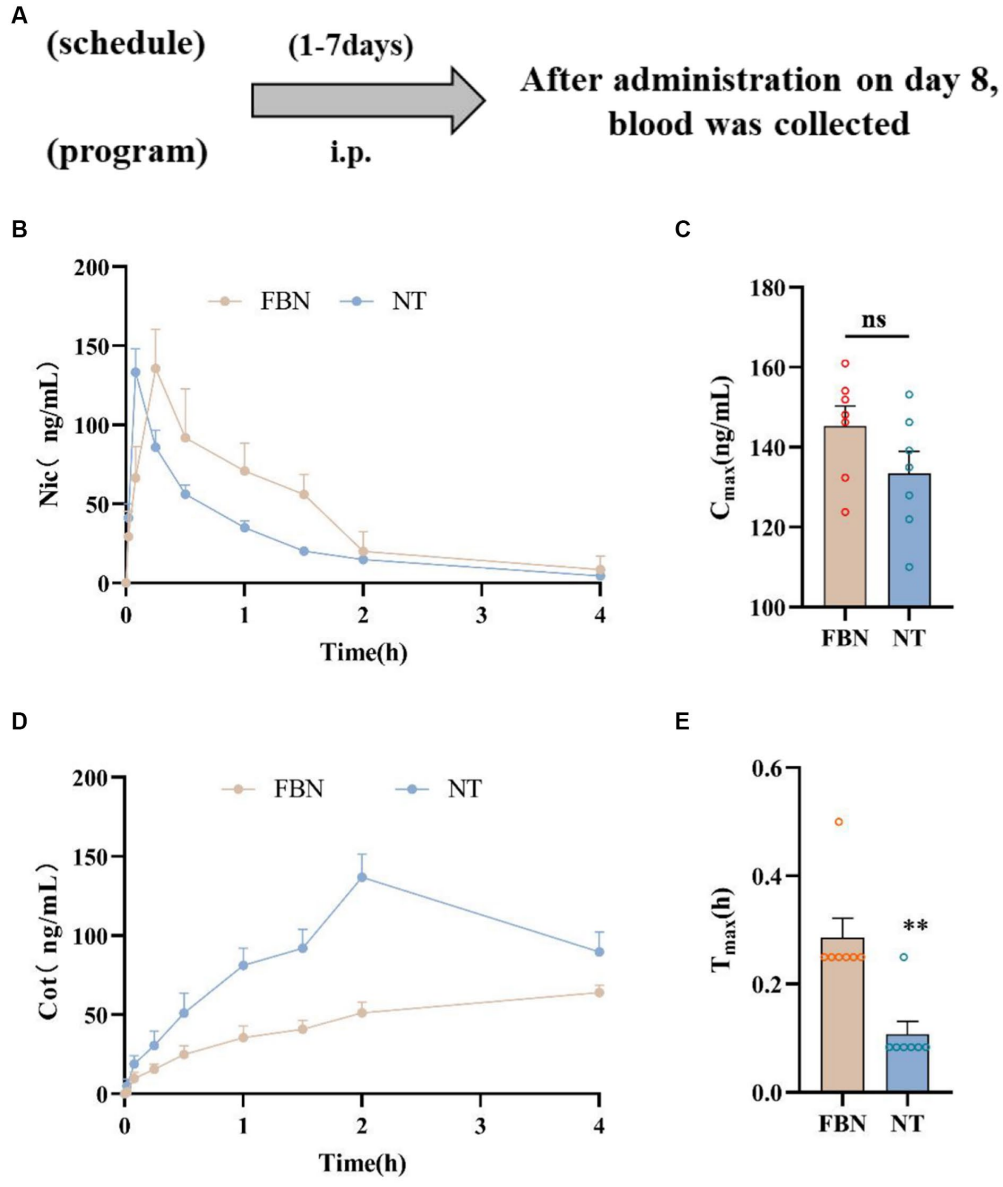
Pharmacokinetic curves of plasma nicotine levels in rats treated with two different types of nicotine. **(A)** Timeline of pharmacokinetic experiments. **(B)** Mean plasma concentration-time plot of nicotine after intraperitoneal administration of nicotine-freebase (0.15 mg/kg nicotine dose) and nicotine tartrate (0.3 mg/kg nicotine dose). **(C)** Maximum concentration (Cmax) of nicotine after nicotine tartrate and nicotine-freebase administration. **(D)** Mean plasma concentration-time plot of cotinine (Cot) after intraperitoneal administration of nicotine-freebase (0.15 mg/kg nicotine dose) and nicotine tartrate (0.3 mg/kg nicotine dose). **(E)** Time to reach maximum concentration (Tmax) after nicotine tartrate and nicotine-freebase administration. Simple *t*-test: **p < 0.01. All data are presented as Mean ± SEM (*n* = 7).

Experiments depicted in Figure 3A used 14 SD rats that were randomly allocated into two groups (nicotine-freebase and nicotine tartrate), each containing 7 rats. The rats received intraperitoneal injections of either 0.15 mg/kg nicotine or 0.3 mg/kg nicotine tartrate twice daily over 7 days, after which blood samples were collected on the 8th day for analysis. Pharmacokinetic results at the same dosage (0.5 mg/kg) have been previously reported in earlier studies conducted by our group (Han et al., 2022). To understand the pharmacokinetic outcomes at doses relevant to self-administration, we selected doses based on the daily amount of drug acquired by the two groups of rats during the self-administration process (Dukes et al., 2020).

#### 2.1.4. DESI-MSI and RT-qPCR

**Figure 4:**
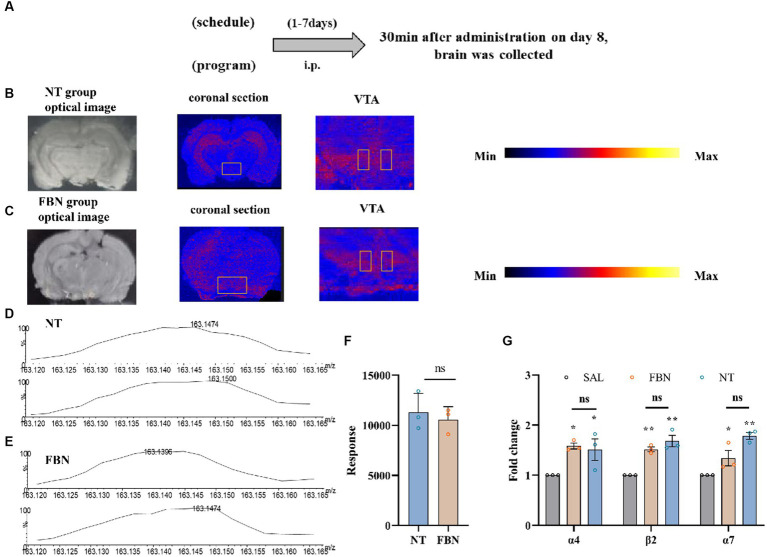
The distribution and content of nicotine in rat brain tissue, as well as its impact on the mRNA expression of three nAChR subunits, following intraperitoneal injection of 0.5 mg/kg nicotine or nicotine salt. **(A)** Timeline of DESI-MSI and RT-qPCR experiments **(B,C)** Coronal sections containing VTA from the nicotine tartrate group and the nicotine-freebase group, with a spatial resolution of 150 μm. Local scans of the VTA in coronal sections of the two groups, with a spatial resolution of 50 μm. **(D,E)** The mass spectra of nicotine in the selected region (VTA). **(F)** Mass spectral response intensities of nicotine in the selected region (VTA) in nicotine-freebase and nicotine tartrate groups, Simple t-test: ns, *p* > 0.05. **(G)** RT-qPCR analysis of fold change in mRNA expression of nAChRs (α4, β2, α7) in the VTA. Bonferroni Student-*t* test after ANOVAS: *p < 0.05, **p < 0.01. All data are presented as Mean ± SEM (*n* = 3).

The DESI-MSI and RT-qPCR experiments depicted in Figure 4A were conducted using 15 SD rats, with 6 randomly assigned to the DESI-MSI experiment and randomly divided into two groups (nicotine-freebase and nicotine tartrate) with 3 rats in each group. The remaining 9 rats needed for RT-qPCR were randomly divided into three groups (saline, nicotine-freebase, and nicotine tartrate), with 3 rats in each group. Before the start of the two experiments, rats in the nicotine-freebase or nicotine tartrate groups received intraperitoneal injections of either 0.5 mg/kg nicotine-freebase or 0.5 mg/kg nicotine tartrate twice daily for 7 days. On the 8th day, brain tissue was collected 30 min after injection.

Brain tissue from the DESI-MSI experiment was embedded in pure water, frozen at −80°C, and cut into 10 μm thick slices using a cryogenic microtome. The slices were mounted on glass slides and stored at −80°C until the beginning of the experiment. The brain tissue in RT-qPCR experiments was placed in an ice-water mixture to extract VTA from rats. After removing blood and impurities, tissues were immediately stored in liquid nitrogen. All VTA samples were stored at −80°C until the beginning of the experiment.

#### 2.1.5. DA release after a single administration of the same dose

**Figure 5:**
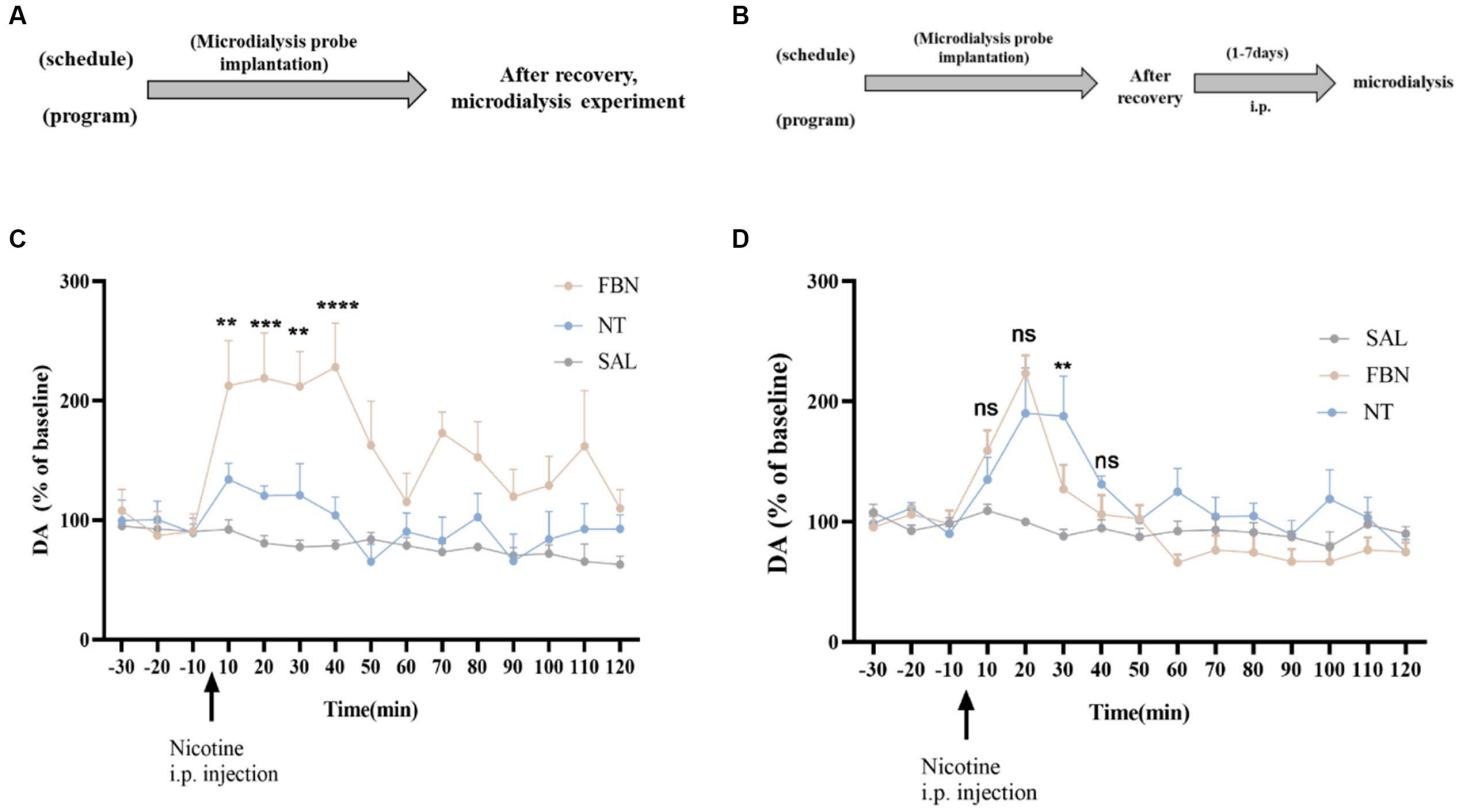
**(A,B)** Timeline of microdialysis experiments **(C)** Time-course effects of nicotine-freebase and nicotine tartrate on the extracellular level of DA in the nucleus accumbens after a single administration at a dose of 0.5 mg/kg. The release of DA in nicotine tartrate group was significantly higher than that in nicotine-freebase group within 10 min-40 min after injection [Two-way ANOVA, *F* (14, 225) = 3.188, *p* = 0.0001; **p < 0.01, ***p < 0.0001, **p < 0.01 and ****p < 0.0001]. **(D)** Time course effects of nicotine-freebase(0.5 mg/kg) and nicotine tartrate(1 mg/kg) on DA extracellular levels in the nucleus accumbens. There was no significant difference in the increases in extracellular dopamine between nicotine tartrate group and nicotine-freebase group at 10–20 min after injection [Two-way ANOVA, *F* (2, 225) = 29.68]. Percentages relative to baseline were calculated from the three samples preceding the treatment administration. Nicotine-freebase (0.5 mg/kg) and nicotine tartrate (1.0 mg/kg) were injected intraperitoneally after baseline measurement (see arrow). Bonferroni Student-t test after ANOVAS: **p < 0.01, and ***p < 0.001 versus FBN group. All data are presented as Mean ± SEM (*n* = 6).

The experiments illustrated in Figure 5A involved 24 SD rats randomly divided into three groups (saline, nicotine-freebase, nicotine tartrate), each containing 8 rats. The drugs (nicotine-freebase and nicotine tartrate) were administered at a dose of 0.5 mg/kg (calculated as free base nicotine) throughout microdialysis experiments, the dosage selection is based on previous research (Nisell et al., 1997; Zhang et al., 2009).

#### 2.1.6. DA release after continuous administration of different doses

The experiments depicted in Figure 5B involved 24 SD rats randomly assigned to three groups (saline, nicotine-freebase, nicotine tartrate), each containing 8 rats. After a recovery period following surgery, rats received intraperitoneal injections of 0.5 mg/kg nicotine or 1 mg/kg nicotine tartrate twice daily. The choice of dosage is based on self-administered experimental results nicotine salt required approximately twice as many infusions as free-base nicotine at the same dose. On the 8th day, microdialysis experiments were performed.

### 2.2. Animals

Male Sprague–Dawley (SD) rats (6–7 weeks) were acquired from Beijing Vital River Laboratory Animal Technology Co., Ltd. After arrival, rats had a week adaptation period, during which time rats were housed in individual cages with access to food and water *ad libitum* and were maintained on a 12-h light/dark cycle. After the experiment began, the weight of rats was controlled. Under experimental conditions, rats received 20 g of food daily, maintained unrestricted access to water, and received a 12-h light, 12-h dark cycle. Tests were carried out during the dark phase of the light/dark cycle. Experiments were conducted according to the National Institutes of Health Guide for the Care and Use of Laboratory Animals. The procedures were approved by the Laboratory Animal Management and Ethics Committee of China National Tobacco Quality Supervision and Test Center.

### 2.3. Drugs

Nicotine-freebase with a purity exceeding 99% was acquired from the China National Tobacco Quality Supervision and Test Center. Benzoic acid and tartaric acid were acquired from Alfa Aesar (Heysham, UK), while lactic acid was acquired from Aladdin (Shanghai, China).

Nicotine benzoate (NB), nicotine lactate (NL), and nicotine tartrate (NT) were prepared by dissolving nicotine-freebase and the corresponding organic acid in 0.9% sterile saline with a molar ratio of organic acid: nicotine-freebase = 2:1. All solutions are prepared fresh daily. Both nicotine salt and nicotine-freebase doses are presented as nicotine-freebase.

### 2.4. Surgery

Jugular vein cannulation was performed as previously described (Harms and Ojeda, 1974). Prior to animal surgery, all surgical instruments were disinfected using 75% ethanol and exposed to UV light for 30 min. Rats were anesthetized using 5% isoflurane gas and injected with Zoletil™ 50 (Virbac) intramuscularly. After complete anesthesia, the hair on the right chest and part of the back of the rat was removed using an electric shaver. During the surgery, rats were fixed in a supine position on the operating table, and the exposed skin was disinfected with iodine and the jugular vein was found in the right chest portion of the rat, and venous vessels were isolated. A small opening was cut on the telecentric end, and a pre-sterilized silicone tube was inserted into the vein through the incision. After the silicone tube was secured, the other end of the silicone tube was connected to the self-administration button (Instech, VABR1B/22) located on the rat’s back. After the surgery, the rats received daily intravenous injections (IV) of saline containing heparin and antibiotics. Rats were given at least 7 days to recover prior to the self-administration test.

### 2.5. Operant responses to sucrose self-administration

According to previous research, food training can make rats learn to touch active nose-poke before self-administration experiments of drugs (Kazan and Charntikov, 2019; Marusich et al., 2019; Carreno and Lotfipour, 2023). Sucrose training and nicotine self-administration sessions were conducted as previously described (Stairs et al., 2007). Two nose-poke operandi were available in the chambers, with nose pokes in one (active) operandum resulting in access to 0.2 ml of a 10% sucrose solution. The chamber light would then turn off for 10 s while a beeping sound played for 1 s. Nose pokes in the other (inactive) operandum were recorded, but had no effect. The experiments were conducted using a fixed ratio (FR) schedule, limited to a maximum of 50 sucrose intakes per day for 5 days, progressing from FR1 training to the end of FR3 (FR1, FR2, FR3, touching 1, 2, 3 times in order to receive a reward). Rats were initially trained to respond on an FR1 schedule of reinforcement, and once 50 rewards were received, they progressed to FR2, and likewise, FR3. Rats that failed to learn the nose-poke response for reward after 5 days of training (<40 FR3 rewards) were excluded from the study.

### 2.6. Operant responding for nicotine self-administration

The protocol for nicotine self-administration was similar to the one used for sucrose, as described above. Experiments were carried out after at least 7 days to allow for recovery from surgery.

As the animals completed sucrose training in the FR3 sessions prior to the surgery, testing began directly using the FR3 schedule, 1 h at a time for 14 days.

### 2.7. Open field test

The OFT was used to evaluate locomotor activity and anxiety-like behavior (Haider et al., 2019; Kaur et al., 2019; Yunusoğlu, 2021; Miao et al., 2023). Before testing, rats were put into the experimental environment for 30 min to acclimate to it. During the test, rats were placed individually in the center of an open field and allowed to freely explore the area for 15 min. The central area of the open field was defined by a 60 cm × 60 cm area. An overhanging camera linked to a computer was used to monitor the rats’ behavior. The arena was cleaned with 75% alcohol between trials to ensure that previous rats’ imprints did not influence subsequent rat behavior. Video tracking and data analysis were performed using Smart video tracking software 3.0.

### 2.8. Elevated plus maze

The elevated plus-maze test was used to measure anxiety-like behavior and was carried out as previously reported (Levin et al., 2019; Miao et al., 2023).

Before testing, rats were put into the experimental environment for 30 min to acclimate to it. The elevated plus maze was composed of a gray wooden cross, elevated 70 cm above the ground, with four arms (90 cm × 8 cm) - two of which were open while the other two were enclosed by side walls (10 cm high). The rats were placed in the center of the intersection of the four arms, facing an open arm. Their activity was monitored by a suspended camera connected to a computer. To avoid any impact on subsequent rat behavior, the maze was cleaned with 75% alcohol between trials, ensuring the removal of any imprints left by previous rats. Video tracking and data analysis were performed using Smart video tracking software 3.0.

### 2.9. *In vivo* nicotine pharmacokinetics

After intraperitoneal injection of drugs (0.15 mg/kg nicotine-freebase or 0.3 mg/kg nicotine tartrate,) for 7 consecutive days, blood collection was performed on the 8th day. After intraperitoneal injection of 0.15 mg/kg nicotine-freebase or 0.3 mg/kg nicotine tartrate, blood samples (0.2–0.4 ml each) were collected from the rat jugular vein at 0, 5, 10, 15, 20, 30, 60, 120, and 240 min. Place the sample in a 1.5 ml centrifuge tube containing 20 μl of heparin sodium. After collection, immediately place the blood sample on ice, followed by centrifugation at 3,500 × *g* for 10 min. Collect the supernatant and freeze it at −80°C for further analysis. Prior to analysis, add 50 μl of internal standard solution and 350 μl of methanol to a 100 μl sample, then vortex, centrifuge, transfer to a chromatographic analysis vial, and proceed with the injection analysis.

The plasma nicotine concentration was measured using an ultra-high performance liquid chromatography–tandem mass spectrometry (UPLC-MS/MS) method, which has been previously described (Liu et al., 2019; Han et al., 2022). The optimized chromatographic conditions are as follows, complete column separation was performed on an ACQUITY UPLC-BEH-HILIC HPLC column (Waters, 2.1 × 150 mm, 1.7 μm), and the column temperature was 40°C. The mobile phase consisted of 10 mmol/L ammonium formate (pH = 3.5) (A) and pure acetonitrile (B) at a flow rate of 0.7 ml/min. Injection volume 1 μl; Running time 10 min.

### 2.10. Desi-MSI

All MSI experiments were conducted on a Xevo G2-XS Q-TOF instrument (Waters, Milford, MA, USA). 10 μm samples on glass slides were subjected to DESI-MSI in positive ion mode, spanning a mass range of m/z 50 to 600. The spray solvent was composed of 85% methanol, 15% H2O, 0.1% formic acid, and 400 ng/ml leucine enkephalin, flowing at 2 μl/min and 0.45 MPa pressure. Source parameters were set at 4.0 kV capillary voltage and 150°C source temperature. DESI-MS images were acquired by raster-scanning tissues at 300 μm/s or 150 μm/s velocities, with spatial resolutions of 150 μm or 50 μm, respectively. The mass spectral data were processed and two-dimensional spatially resolved ion images were generated using the high-definition imaging platform version 1.6 (Waters).

### 2.11. Quantitative real-time polymerase chain reaction

The RT-qPCR was performed using a RT-qPCR kit (Accurate Biology), following the instructions provided by the manufacturer.

### 2.12. Microdialysis and dopamine detection

Microdialysis experiments were performed as previously described (Chefer et al., 2009). Rats were anesthetized using 5% isoflurane gas and injected intramuscularly with Zoletil™ 50 (Virbac). After complete anesthesia, an electric shaver was used to remove hair beginning from the region between the eyes to the posterior portion of the skull. Surgery was performed using aseptic technique, and microdialysis cannulae and probes were sterilized using UV light. Rat heads were fixed on a stereotactic apparatus. According to the rat brain stereotaxic reference book (Paxinos and Watson, 2006), the position of the nucleus accumbens shell was established as anterioposterior (AP): +2.5 mm, mediolateral (ML): +1.4 mm, and dorsoventral (DV): −7.0 mm. After surgery, rats were allowed to recover for 3–5 days. Microdialysis experiments were divided into single-dose and multiple-dose administrations. Single-dose administration was carried out on the day of the microdialysis experiment with a single dose of 0.5 mg/kg nicotine-freebase or nicotine tartrate, followed by sample collection. Multiple-dose administration involved exposing the animals to nicotine-freebase (0.5 mg/kg) or nicotine tartrate (1 mg/kg) via intraperitoneal injection for 7 days prior to the microdialysis experiment. One additional intraperitoneal injection was administered before sample collection. On the day of microdialysis, the rats were connected to a microdialysis instrument for 2 h at a flow rate of 1.0 μl/min to maintain a stable baseline. After adaptation, the microdialysis flow rate was set at 2.0 μl/min and samples were collected every 10 min. Each rat was measured at 15 time points, with the first 3 time points used as baseline values, with the drug injected beginning at the 4th time point. The detection of dopamine used high-performance liquid chromatography combined with an electrochemical detector (HPLC-ECD).

### 2.13. Statistical analysis

The analysis of data was performed through one-way analysis of variance (ANOVA), two-way ANOVA, and t-tests when appropriate using GraphPad Prism 8.0 software. For comparisons between only two groups, an unpaired t-test was used to calculate the *p*-value. For comparisons between more than two groups, one-way ANOVA was used, and when there were multiple independent variables, two-way ANOVA was used. Following ANOVAs, Bonferroni-corrected t-tests were performed for *post hoc* comparisons. The results were presented as mean ± SEM in the figures. Statistical significance was set at *p < 0.05, **p < 0.01, ***p < 0.001, and ****p < 0.0001.

## 3. Results

### 3.1. Nicotine self-administration

Nicotine-freebase and nicotine salts self-administration in rats was performed according to the experimental schedule illustrated in Figure 1A. The amount of drug delivered during self-administration (1 h per day, 14 days) was controlled by a fixed Ratio 3 (FR3) schedule as shown in Figure 1B. In the last 4 days of self-administration under the FR3 schedule, the average number of infusions in each group is shown in Figure 1C. Compared to the saline group, the nicotine-freebase group (^++++^*p* < 0.0001), nicotine tartrate group (^++++^*p* < 0.0001), nicotine benzoate group (^++++^*p* < 0.0001), and nicotine lactate group (^++++^*p* < 0.0001) all had a significantly higher amount of drug delivery. Rats assigned the nicotine salts earned a significantly higher number of FR3 deliveries compared to nicotine-freebase [*F* (3, 12) = 68.76, ****p < 0.0001]. However, a one-way analysis of variance (ANOVA) demonstrated no significant difference in drug delivery times among three groups of nicotine salts self-administered rats [*F* (2, 9) = 3.835, ns, *p* > 0.05]. In addition, the results of self-administration of three organic acids without nicotine showed that the individual organic acids did not produce reinforcement effects (Supplementary Figure S1). Meanwhile, the impact of pH on rats’ self-administration of nicotine tartrate was also examined. It was observed that pH 7.4 (0.03 mg/kg, nicotine tartrate) and pH 3.5 (0.03 mg/kg, nicotine tartrate, unadjusted pH) induced similar drug delivery (Supplementary Figure S2).

### 3.2. Withdrawal-related behavioral testing after self-administration

**Figure 6:**
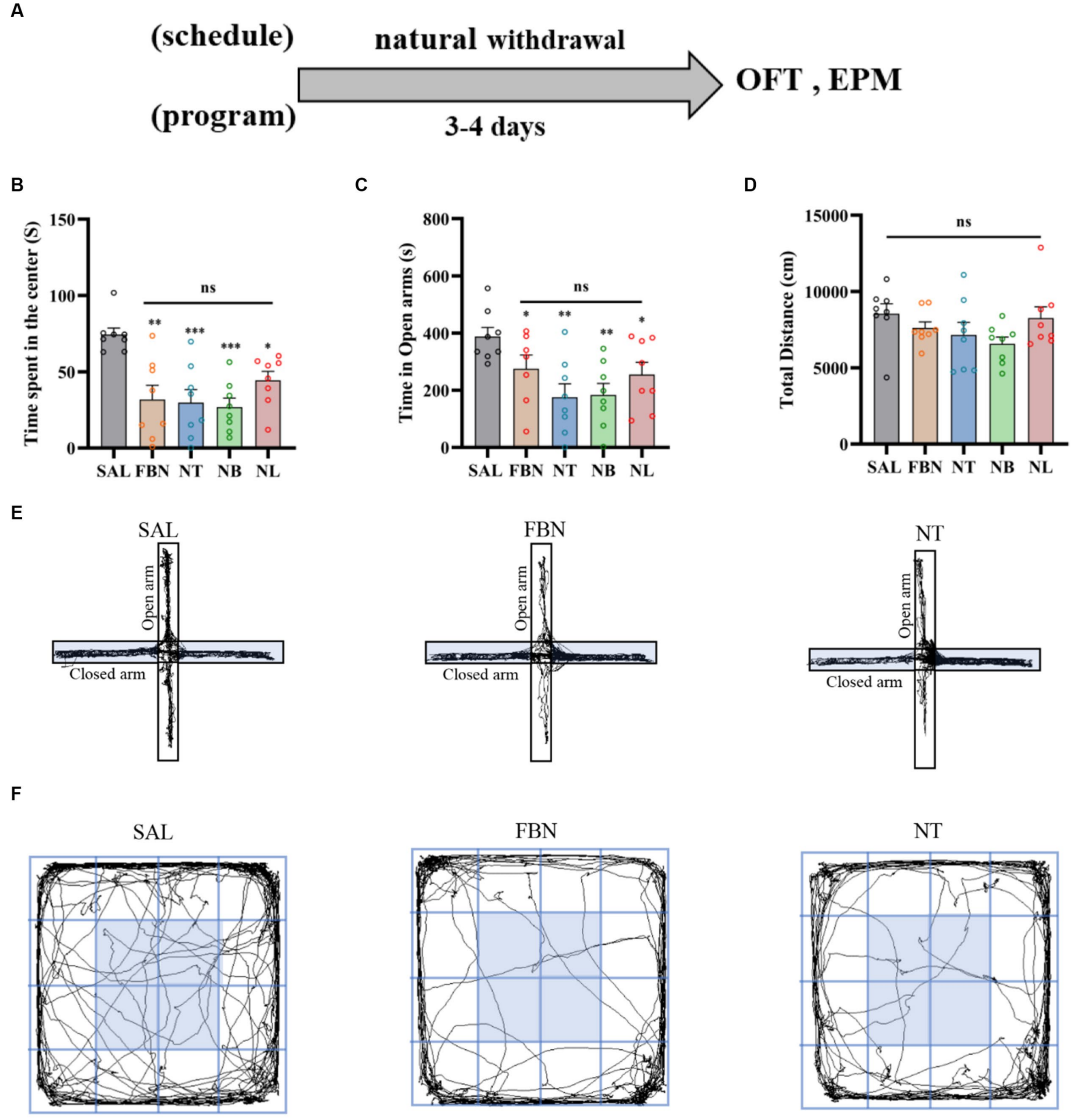
The elevated plus maze (EPM) and open field test (OFT) were conducted 3–4 days after the end of self-administration and natural withdrawal. **(A)** Timeline of withdrawal-related behavioral experiments. **(B)** Time spent in the center. **(C)** Open arm duration. **(D)** Total distance traveled. **(E)** Representative images of each group in the EPM. **(F)** Representative images of each group in the OFT. Only nicotine tartrate group of representative images is displayed for the nicotine salt groups, as there were no significant differences in withdrawal-related behaviors among these groups. Bonferroni Student-*t* test after ANOVAS: *p < 0.05, **p < 0.01, and ***p < 0.001. All data are presented as Mean ± SEM (*n* = 8).

Three days after the end of self-administration, we conducted the Elevated Plus Maze (EPM) and Open Field Test (OFT) to assess withdrawal reactions in rats exposed to either nicotine or nicotine salt. Results from the OFT indicated that when compared to the saline group [*F* (4, 35) = 7.842; *p* = 0.001; Figure 6B], the time spent in the center was significantly lower in the nicotine-freebase group (*p < 0.05), nicotine tartrate group (**p < 0.01), nicotine benzoate group (**p < 0.01), and nicotine lactate group (*p < 0.05). There were no significant differences in the time spent in the center between the three nicotine salts groups and the nicotine-freebase group [*F* (3, 28) = 1.061; ns, *p* > 0.05; Figure 6B], and no changes were observed in the motor ability of rats in each group [*F* (4, 36) = 1.637; ns, *p* > 0.05; Figure 6D].

With respect to the EPM, when compared to the saline group [*F* (4, 34) = 4.253, *p* < 0.01], the open arm duration was significantly lower in the nicotine-freebase group (**p < 0.01), nicotine tartrate group (***p < 0.001), nicotine benzoate group (***p < 0.001), and nicotine lactate group (*p < 0.05). There were no significant differences in the open arm duration between the three nicotine salts groups and the nicotine-freebase group [*F* (3, 27) = 1.251; ns, *p* > 0.05; Figure 6C].

### 3.3. Withdrawal-related behaviors under identical dose administration

Before conducting the OFT and EPM experiments, animals received drug injections twice daily for seven consecutive days, followed by a natural withdrawal period lasting 3–4 days. The results indicated that when compared to the withdrawal-related behavioral tests following self-administration, anxiety symptoms were lower in the three nicotine salts groups than in the nicotine-freebase group (Figure 2).

The OFT results indicated that when compared to the saline group [*F* (4, 35) = 39.31; *p* < 0.0001; Figure 2B], the time spent in the center was significantly lower in the nicotine-freebase group (^++++^*p* < 0.0001), nicotine tartrate group (^++++^*p* < 0.0001), nicotine benzoate group (^++++^*p* < 0.0001), and nicotine lactate group (^++++^*p* < 0.0001). In comparison with the nicotine-freebase group [*F* (3, 28) = 14.25, *p* < 0.0001], the time spent in the center in nicotine tartrate group (***p < 0.001), nicotine benzoate group (**p < 0.01) and nicotine lactate group (*p < 0.05) was significantly higher than those in the nicotine-freebase group (Figure 2B). There were no significant differences in the time spent in the center across the three nicotine salts groups [*F* (2, 21) = 4.574; ns, *p* > 0.05; Figure 2B]. The motor ability of rats in each group did not change in general [*F* (4, 42) = 4.688; ns, *p* > 0.05; Figure 2D].

The EPM results indicated that when compared to the saline group [*F* (4, 35) = 34.43; *p* < 0.0001], the open arm duration was significantly lower in the nicotine-freebase group (^++++^*p* < 0.0001), nicotine tartrate group (^++++^*p* < 0.0001), nicotine benzoate group (^++++^*p* < 0.0001), and nicotine lactate group (^++++^*p* < 0.0001). In comparison with the nicotine-freebase group [*F* (3, 28) = 13.56, *p* < 0.0001], the open arm duration in nicotine tartrate group (**p < 0.01), nicotine benzoate group (**p < 0.01) and nicotine lactate group (***p < 0.001) were significantly higher than those in the nicotine-freebase group (Figure 2C).

### 3.4. Pharmacokinetics of self-administration related doses

The results demonstrated that nicotine was rapidly absorbed and distributed in rats following Intraperitoneal injection (Figure 3). The concentration of nicotine peaked after 5–15 min (Figures 3B,E). No significant difference was observed in nicotine Cmax between the nicotine-freebase and nicotine tartrate groups (double the dose of the nicotine-freebase group; ns, *p* > 0.05; Figure 3C). However, there was a significant difference in Tmax between the nicotine tartrate groups and nicotine-freebase group (**p < 0.01; Figure 3E), suggesting that the nicotine tartrate reached Cmax more rapidly. In addition, plasma cotinine levels were higher in the nicotine tartrate group than in the nicotine-free base group (Figure 3D). The detailed pharmacokinetic parameters were shown in Supplementary Table S1.

### 3.5. DESI-MSI

All MSI experiments were conducted on a Xevo G2-XS Q-TOF instrument (Waters, Milford, MA, USA). 10 μm samples on glass slides were subjected to DESI-MSI in positive ion mode, spanning a mass range of m/z 50 to 600. The spray solvent was composed of 85% methanol, 15% H2O, 0.1% formic acid, and 400 ng/ml leucine enkephalin, flowing at 2 μl/min and 0.45 MPa pressure. Source parameters were set at 4.0 kV capillary voltage and 150°C source temperature. DESI-MS imaging was used to visualize spatial distribution of nicotine throughout rat brain tissue (Figure 4). Ion images demonstrated that in the nicotine tartrate group, nicotine (m/z 163.124) was mainly distributed in the hippocampus. In comparison, nicotine (m/z 163.124) distribution in the nicotine-freebase group was more scattered (Figures 4B,C). However, there was no significant difference in the distribution of nicotine within the VTA of the two groups (ns, *p* > 0.05; Figures 4D–F).

### 3.6. Expression of α4, β2, and α7 nAChR subunit mRNA in VTA

The RT-qPCR experiments (Figure 4G; α4, *F* (2, 6) = 6.159, *p* < 0.05; α7, F (2, 6) = 16.44, *p* < 0.01; β2, F (2, 6) = 25.18, *p* < 0.05) revealed that compared to the saline group, the mRNA expression of α4 (*p < 0.05), β2 (**p < 0.01), and α7 (*p < 0.05) nicotinic acetylcholine receptors (nAChRs) are significantly increased in the nicotine-freebase group, and α4 (*p < 0.05), β2 (**p < 0.01), and α7 (**p < 0.01) nAChRs significantly increased in the nicotine tartrate group. However, there was no significant difference in nAChRs mRNA expression between the nicotine-freebase and nicotine tartrate groups (ns, *p* > 0.05).

### 3.7. Dopamine detection

After a single intraperitoneal administration (0.5 mg/kg nicotine-freebase, 0.5 mg/kg nicotine tartrate), the results of microdialysis experiments demonstrated (Figure 5C) a marked increase in extracellular DA release for both the nicotine-freebase and nicotine tartrate groups compared with the saline group. Following intraperitoneal injection of 0.5 mg/kg nicotine-freebase, the peak DA release was reached between 10 to 40 min, approximately 220%. After intraperitoneal injection of 0.5 mg/kg nicotine tartrate, the peak DA release occurred between 10 to 40 min, approximately 140%. The increase in extracellular dopamine was more significant in the nicotine-freebase group compared to the nicotine tartrate group, with a significant difference in the peak values (**p < 0.01, ***p < 0.0001, **p < 0.01, and ****p < 0.0001).

After continuous administration for 7 days at different doses (0.5 mg/kg nicotine-freebase, 1 mg/kg nicotine tartrate), the results of microdialysis experiments demonstrated (Figure 5D) a marked increase in extracellular DA release for both the nicotine-freebase and nicotine tartrate groups compared with the saline group. Following intraperitoneal injection of 0.5 mg/kg nicotine-freebase, the peak DA release was observed only at 20 min, approximately 220%. Following intraperitoneal injection of 1 mg/kg nicotine tartrate, the peak DA release occurred between 20 to 30 min, approximately 190%. Compared to the nicotine tartrate group, there was a significant difference in extracellular dopamine increase only at 30 min in the nicotine-freebase group (**p < 0.01), with the overall DA release being relatively similar between the two groups.

### 3.8. Correlation analyses between pharmacokinetics and addictive behavior

**Figure 7:**
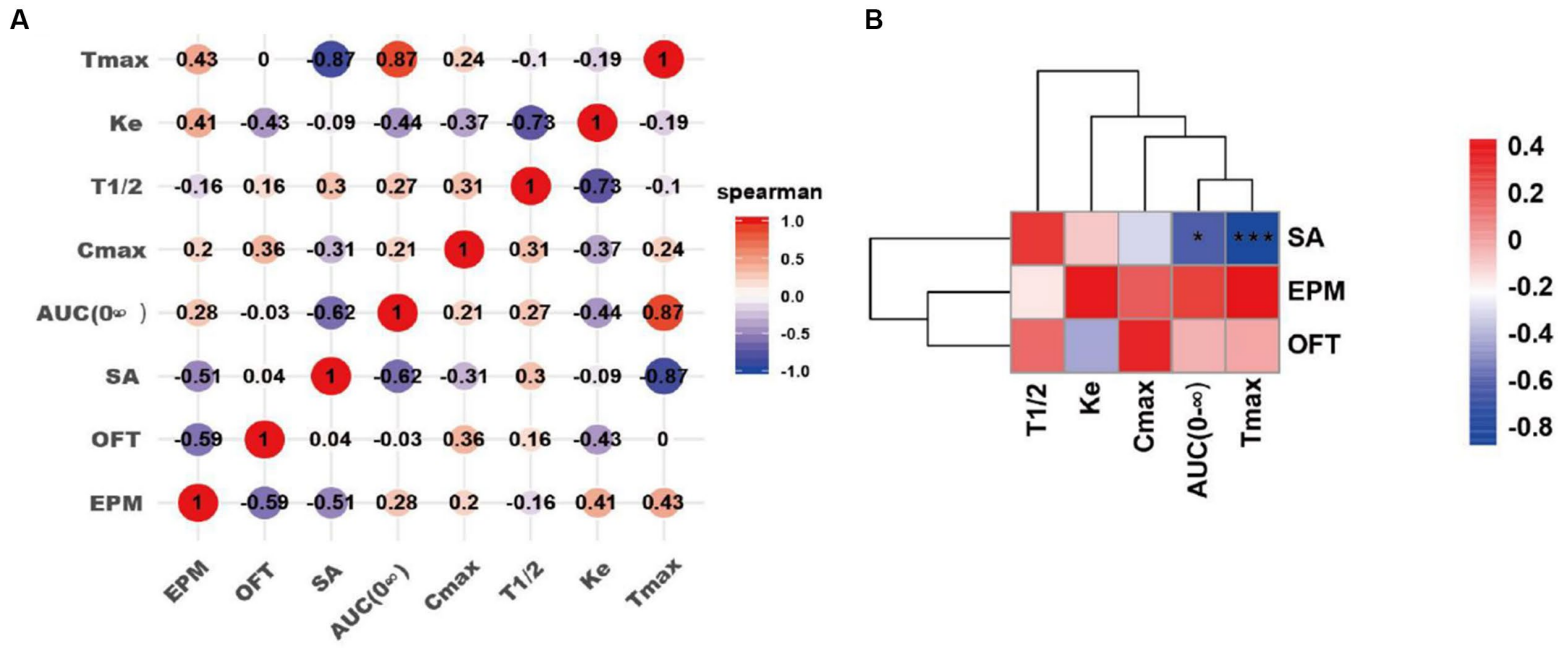
Correlation analyses of pharmacokinetics of the drug self-administrated and addictive behaviors. **(A)** Correlation matrix diagram. **(B)** Correlation clustering heat map. SA: the number of infusions in nicotine self-administration (SA) experiments; OFT: the time spent in the center of rats in the OFT 3–4 days after nicotine withdrawal; EPM: the time in the open arms of rats in the EPM 3–4 days after nicotine withdrawal. Correlation algorithm: spearman. *p < 0.05, and ***p < 0.001 (Correlation significance).

To investigate the impact of pharmacokinetics (Cmax, Tmax, AUC, T1/2 and Ke) of nicotine in the addictive behaviors, we have performed the linear correlation analysis among the parameters of pharmacokinetics and behaviors. Our data indicated that Tmax and AUC were significantly negatively correlated with infusion number of SA test (AUC, *p < 0.05; Tmax, ***p < 0.001). However, no significant correlations were observed between pharmacokinetics (Cmax, T1/2 and Ke) and infusion number of SA test. No significant correlations were observed between pharmacokinetics (Cmax, Tmax, AUC, T1/2 and Ke) and time spent in the center in the OFT, as well as these parameters and time in the open arms of EPM after nicotine SA withdrawal (Figure 7).

## 4. Discussion

This study aimed to compare the reinforcing effects of nicotine-freebase and nicotine salts in animal models of drug self-administration and investigates whether pharmacokinetic induced these differences. Additionally, the study aimed to explore potential neurobiological mechanisms that contribute to these differences. Our results showed that, first, at the same dosage, compared with nicotine-freebase, nicotine salts resulted in greater drug delivery, while nicotine-freebase caused stronger withdrawal-induced anxiety symptoms and a greater extracellular dopamine increase in the NAc. There were no significant differences in nicotine distribution and expression of α4, β2, and α7 nAChR subunit mRNA in the VTA between the nicotine-freebase and nicotine tartrate groups. Second, at self-administration relevant dosages, the peak plasma nicotine concentrations were similar between the nicotine-freebase group (0.15 mg/kg) and the nicotine tartrate group (0.3 mg/kg), with a shorter peak time in the nicotine tartrate group. The extracellular DA increase in the NAc was similar between the nicotine-freebase group (0.5 mg/kg) and the nicotine tartrate group (1 mg/kg). These findings suggest that nicotine-freebase may be more potent than nicotine salts, and the pharmacokinetic differences between nicotine-freebase and nicotine salts might lead to differences in extracellular DA increases in the NAc, thereby affecting behavioral differences.

Reinforcement-related behavior experiments allowed animals to associate specific operant behaviors with rewards in drug research, thereby simulating human drug abuse behavior (Donny et al., 1998).

Our results demonstrate that at the beginning of the self-administration experiment, each group of rats maintained a relatively high level of drug infusion, which might be due to the rats receiving training on sucrose solution prior to nicotine self-administration, resulting in a desire for sucrose solution at the start of nicotine self-administration. This finding is consistent with previous research. This phenomenon rapidly disappeared within 3 to 4 days when the response on the active nose-poke did not result in sucrose solution delivery (Marusich et al., 2019; Carreno and Lotfipour, 2023). The results of nicotine self-administration showed that nicotine salts (nicotine tartrate, nicotine benzoate, nicotine lactate) and nicotine-freebase induced reinforcement-related behaviors in rats compared with saline. However, nicotine salts (nicotine tartrate, nicotine benzoate, nicotine lactate) produced greater reinforcement-related behaviors in rats when compared to nicotine-freebase, with drug delivery in the nicotine salts group being approximately twice that of the nicotine-freebase group over the final 4 days, and no significant differences were observed across the three nicotine salts groups. In a previous study, Brandon J. et al. observed that in a vapor self-administration model, mice obtained more nicotine tartrate delivery than nicotine-freebase, which is consistent with our observations (Henderson and Cooper, 2021). In addition to nicotine tartrate for reinforcement-related behavior, we also conducted reinforcement-related behavior experiments on two other nicotine salts (nicotine benzoate, nicotine lactate) commonly used in electronic nicotine delivery systems (ENDS) and explored the potential mechanism to understand the difference in reinforcement-related behavior between nicotine salts and nicotine-freebase.

Withdrawal-related behaviors were an important aspect of nicotine dependence (Alsharari et al., 2015). In the rodent model, nicotine withdrawal reduced the residence time of animals in the open arm of EPM, the number of times they entered the open arm of the EPM, time in the central area of OFT, and the number of times they entered the central area of OFT (Chellian et al., 2021). Overall, these behaviors indicated an increase in anxiety-like behaviors during withdrawal.

Nicotine-freebase and nicotine salts both caused withdrawal-related anxiety symptoms under the two administration methods, which was consistent with previous findings (Chellian et al., 2021). Interestingly, the withdrawal-related anxiety symptoms were lower in the nicotine salt group than in the nicotine-freebase group.

In animal models, the rate of drug metabolism has been shown to influence reinforcement-related behavior (Caraballo et al., 2011; Krebs et al., 2016; Chen et al., 2020). At a dose of 1 mg/kg of nicotine, our research group found that the peak plasma nicotine concentration of nicotine-freebase was approximately 2 times higher than that of nicotine salts, and the time to reach the peak concentration of nicotine salts (nicotine tartrate, nicotine benzoate, nicotine lactate) was shorter than that of nicotine-freebase (Han et al., 2022). In our investigation, the daily dose of the nicotine salts group was nearly double that of the nicotine-freebase group. Previous findings demonstrate that new popular e-cigarettes (using nicotine salts) contain more nicotine than conventional e-cigarettes (using nicotine-freebase; Han et al., 2016). Therefore, we speculated upon the difference in reinforcement-related behavior and withdrawal behavior and whether it was attributable to the difference in pharmacokinetics of nicotine-freebase and nicotine salts *in vivo*. Previous reports made similar speculations. Brandon J. et al. found that nicotine tartrate resulted in higher drug delivery than nicotine-freebase in a vapor self-administration model and that plasma cotinine levels in the nicotine salts group were greater than that in the nicotine-freebase group under the same exposure. As a result, Brandon J. et al. assumed that the observed differences in reinforcement-related behavior were due to differences in the pharmacokinetics of nicotine-freebase/nicotine salts.

Based upon our speculation, we conducted the several experiments. The study found that the peak plasma nicotine concentration was similar when administered at 0.3 mg/kg nicotine tartrate and 0.15 mg/kg nicotine-freebase, and the time to reach the peak of nicotine salt was shorter than that of nicotine-freebase, which was consistent with previous research. For example, under the same dosage (1 mg/kg), nicotine plasma concentrations produced by nicotine salt were lower than those produced by nicotine-freebase, possibly because nicotine-freebase had higher lipophilicity and was more easily absorbed into the blood, leading to higher concentrations in the plasma. Similarly, nicotine salts reached peak plasma nicotine levels earlier (Han et al., 2022). In an intravenous administration experiment, the researchers found that nicotine-freebase showed higher plasma nicotine levels and a later time to peak than nicotine salts (Hwa Jung et al., 2001). The results of another population experiment showed that all dosages of nicotine lactate salts entered the systemic circulation more quickly than nicotine-freebase (O’Connell et al., 2019).

Nicotine stimulated increases in extracellular dopamine through activation of dopamine neurons, resulting in reward and reinforcement. Microdialysis combined with high-performance liquid chromatography was widely used for monitoring DA and other neurotransmitters in interstitial tissue fluid (Chefer et al., 2009).

Under a single exposure to the drug, both nicotine-freebase and nicotine tartrate groups exhibited peak DA release between 10 to 40 min, with approximately 220 and 130% increases. The results were consistent with the previous reports (Cohen et al., 2002; Azam et al., 2007; Zhang et al., 2018; Sagheddu et al., 2019). Seven days of nicotine acclimation resulted in reduced increases in DA release compared with acute exposure. Following intraperitoneal injection of 0.5 mg/kg nicotine-freebase, the peak DA release was observed only at 20 min, approximately 220%. Following intraperitoneal injection of 1 mg/kg nicotine tartrate, the peak DA release occurred between 20 to 30 min, approximately 190%. Existing literature indicates that repeated exposure to nicotine leads to a reduction in extracellular dopamine (DA) release in the nucleus accumbens. After oral nicotine treatment for several weeks, mice show a decrease in DA release in the NAc (Zhang et al., 2012). Squirrel monkeys, following several months of oral nicotine treatment, also exhibit a reduction in DA release in the NAc (Perez et al., 2012). Additionally, after subcutaneous injection of nicotine for seven consecutive days, it was observed that DA release decreases when compared to the saline control group (Fennell et al., 2020). Overall, the administration of 0.5 mg/kg nicotine-freebase and 1 mg/kg nicotine tartrate could induce similar DA release, potentially due to the comparable plasma nicotine concentrations in both groups at these doses.

We believed that the higher metabolism rate of nicotine salts in the body than nicotine-freebase caused “compensatory behavior,” resulting in behavioral differences. Previous studies reported that lower concentrations of nicotine could cause “compensatory behavior,” with subjects taking longer and deeper puffs more frequently to achieve a similar level of nicotine (Bandiera et al., 2015). Rats with higher nicotine metabolism rates often showed greater compensatory increases (Harris et al., 2008). Faster nicotine metabolizers might have compensated more than slower ones (Ray et al., 2009). Mice with higher plasma nicotine levels may lead to increased anxiety-like behavior (Echeveste Sanchez et al., 2023).

The differences in reinforcement-related behaviors between the nicotine-freebase group and the nicotine salt group were attributed to the lower plasma nicotine levels produced by nicotine salt. Rats in the nicotine salt group need to obtain more drug delivery to achieve similar plasma nicotine levels to nicotine-freebase, leading to a similar increase in extracellular dopamine release in the nucleus accumbens to elicit similar rewarding effects. After self-administration, rats exhibited no significant differences in anxiety-like symptoms. This was because, during the self-administration process, rats acquired drug amounts that resulted in similar plasma nicotine levels. However, after receiving the same dose of the drug, the nicotine-freebase group experienced greater withdrawal-induced anxiety-like symptoms compared to the nicotine salt group. This was attributed to the slower metabolism of nicotine-freebase compared to nicotine salt, resulting in higher plasma nicotine levels.

To further analyze the potential relationship between the pharmacokinetics and addictive behaviors/anxiety during nicotine withdrawal in rats, we performed correlation analyses between the pharmacokinetic indexes (Tmax, AUC (0-∞), Ke, and T1/2) and behavioral parameters including the number of infusions in nicotine SA, time spent in the center in the OFT, time in the open arms of EPM after withdrawal in nicotine-freebase and nicotine tartrate groups. Interestingly, we found a significant negative correlation between the Tmax or AUC (0-∞) of the drug and the addictive behaviors. This indicates that the injection of the drug which has a shorter Tmax or AUC (0-∞) resulted in a reinforcement in addictive behaviors. It has been reported that Tmax and AUC play important roles in the process of drug addiction, and the correlation data is consistent with our current results. Henderson et al. found that in mice SA experiment, non-contingent exposure to nicotine- salt produced higher plasma cotinine values compared to nicotine- freebase (Henderson, et al., 2021); additionally, Salerian A. J. demonstrated a negative correlation between addictive potency and Tmax (Salerian, 2010). Our present data further convinced the relation of pharmacokinetics of nicotine in different chemical forms with addiction-associated behaviors.

We acknowledge that this study had several limitations. Firstly, in the self-administration studies, only a single dose of nicotine (0.03 mg/kg) was administered. Future studies should examine lower and higher doses to determine potential behavioral differences across different nicotine salts. Secondly, the mechanism underlying the metabolic differences between nicotine-freebase and nicotine salts remained unclear, requiring further investigation into mode of delivery, CYP2A6 activity in the liver, and bioavailability. For example, flavoring agents in e-cigarette liquids might alter CYP2A6 activity (Winters et al., 2022). Additionally, this study only used male rats. Previous research showed significant differences in self-administration behavior between male and female rats (Flores et al., 2019; Chellian et al., 2020), and it was necessary to examine behavioral differences in female rats.

In conclusion, our study demonstrated that nicotine salts resulted in stronger reinforcement-related behaviors, whereas nicotine-freebase induced more obvious withdrawal-induced anxiety-like symptoms. The pharmacokinetic profiles of nicotine tartrate (0.3 mg/kg) and nicotine-freebase (0.15 mg/kg) were similar; however, the distribution of nicotine in the brain was different between the nicotine tartrate (0.5 mg/kg) and nicotine-freebase groups (0.5 mg/kg). Nicotine was primarily distributed in the hippocampus in the nicotine tartrate group and more dispersed in the nicotine-freebase group, with no significant differences in nicotine distribution in the VTA. There were no significant differences in mRNA expression of nAChRs between the nicotine-freebase and nicotine tartrate groups (0.5 mg/kg). The nicotine-freebase group exhibited stronger increases in extracellular dopamine than the nicotine tartrate group when receiving the same dose (0.5 mg/kg), while nicotine-freebase (0.5 mg/kg) and nicotine tartrate (1 mg/kg) induced similar increases in extracellular dopamine. Overall, these results indicate that the pharmacokinetic differences between nicotine salts and nicotine-freebase might partially explain observed behavioral differences in rats.

## Data availability statement

The original contributions presented in the study are included in the article/Supplementary material, further inquiries can be directed to the corresponding authors.

## Ethics statement

The animal study was approved by the Laboratory Animal Management and Ethics Committee of China National Tobacco Quality Supervision and Test Center. The study was conducted in accordance with the local legislation and institutional requirements.

## Author contributions

PH: Conceptualization, Data curation, Formal analysis, Investigation, Methodology, Software, Validation, Visualization, Writing – original draft. XJ: Visualization, Writing – original draft. SH: Conceptualization, Supervision, Writing – review & editing. XW: Supervision, Writing – review & editing. QL: Investigation, Writing – review & editing. YZ: Data curation, Investigation, Visualization, Writing – review & editing. PY: Data curation, Investigation, Visualization, Writing – review & editing. X-aL: Writing – review & editing. PW: Writing – review & editing. HC: Funding acquisition, Project administration, Resources, Supervision, Writing – review & editing. HH: Conceptualization, Funding acquisition, Resources, Supervision, Writing – review & editing. QH: Funding acquisition, Resources, Supervision, Writing – review & editing.

## Glossary

**Table 1:** 

E-Cigs	Electronic cigarettes
FBN	Nicotine-freebase
NS	Nicotine salts
NB	Nicotine benzoate
NT	Nicotine tartrate
NL	Nicotine lactate
CPP	Conditioned place preference
DA	Dopamine
OFT	Open field test
EPM	Elevated plus-maze
RT-qPCR	Quantitative real-time polymerase chain reaction
SAL	Saline
SD	Sprague–Dawley
FR	Fixed Ratio
UPLC-MS/MS	Ultra-high performance liquid chromatography–tandem mass spectrometry
AP	Anterioposterior
ML	Mediolateral
DV	Dorsoventral
ANOVA	Analysis of variance
nAChRs	Nicotinic acetylcholine receptors
ENDS	Electronic nicotine delivery systems
AUC	The area under the curve
T1/2	Elimination half-life
Cmax	Maximum plasma concentration
Tmax	The time required to reach the maximum plasma concentration
Ke	Elimination rate constant
