# Supplementary material for: Pharmacokinetic differences in nicotine and nicotine salts mediate reinforcement-related behavior: an animal model study
Source: Front Neurosci. 2023 Nov 16;17:1288102. doi: 10.3389/fnins.2023.1288102 (PMC10687399; doi:10.3389/fnins.2023.1288102)
Supplement: Supplementary file 1 [file Data_Sheet_1.pdf]

Supplementary Material

Pharmacokinetic Differences in Nicotine and Nicotine Salts Mediate Reinforcement-Related Behavior: An Animal Model Study

Pengfei Han *, Huan Chen, Hongwei Hou

*** Correspondence:** Dr. Huan Chen, Dr. Hongwei Hou

Email: hunny_ch@163.com; qsfctc@163.com

1 Supplementary Figures and Tables

1.1 Supplementary Figures

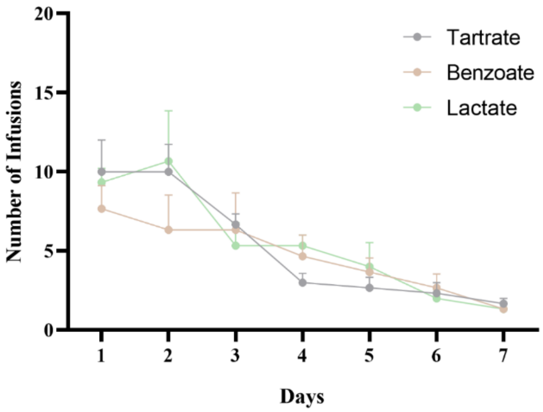

Supplementary Figure 1. Results of organic acid self-administration experiments under a fixed-rate 3 (FR3) schedule. Number of infusions for each group: Tartrate (black circle), Benzoate (yellow circle), Lactate (blue circle). There was no significant difference in the number of infusions among the three organic acid groups. $F(2, 42) = 0.5477$, $p > 0.05$.

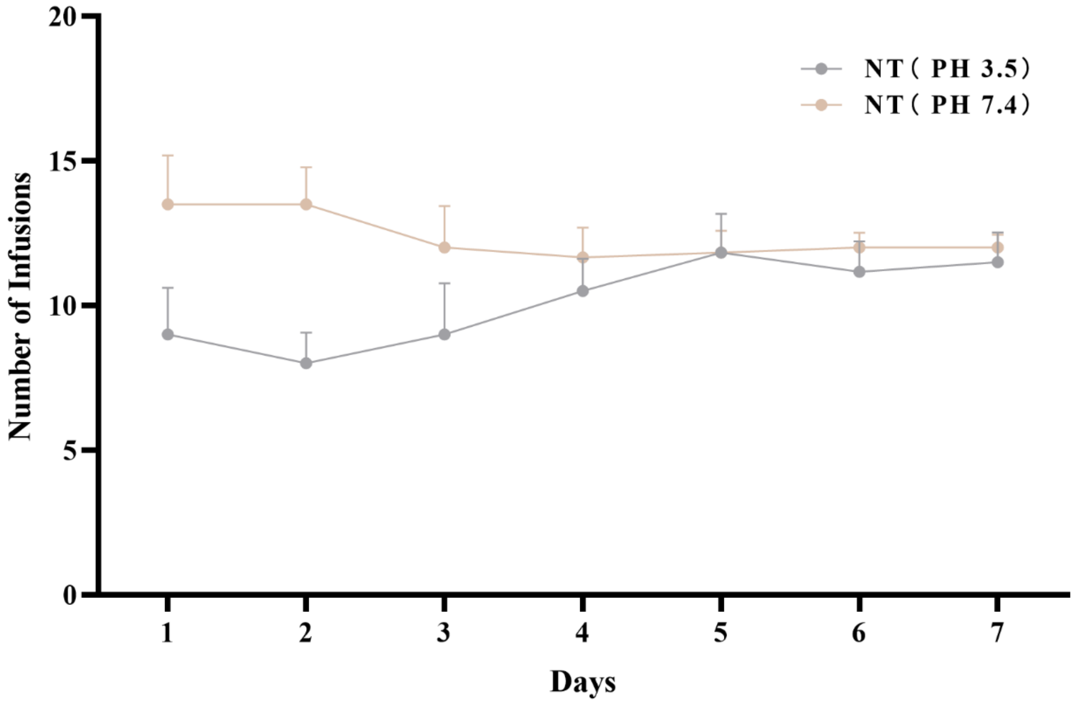

Supplementary Figure 2. The impact of pH on rats' self-administration of nicotine tartrate. Results of self-administration experiments under a fixed-rate 3 (FR3) schedule. Number of infusions for each group: Nicotine tartrate (PH3.5, black circle), Benzoate (PH7.4, yellow circle). $F(6, 70) = 0.1743$, $p > 0.05$.

1.2 Supplementary Tables

Table S1: Mean pharmacokinetic parameters of Nic in rats after administration of FBN and NT.

	**AUC (0-∞)**	**Ke**	**t1/2z**	**Tmax**	**Cmax**
	**ug/L*h**	**1/h**	**h**	**h**	**ug/L**
FBN	184.072 ± 36.712	1.282 ± 1.206	0.921 ± 0.622	0.286 ± 0.094	145.347 ± 12.946
NT	115.377 ± 19.874	0.806 ± 0.351	1.065 ± 0.572	0.083 ±0.062	133.368 ± 14.712

Note: Values are the mean ± SD of six rats

Abbreviations: FBN, nicotine-freebase; NT, nicotine tartrate.
